# Mindset and perceived parental support of autonomy safeguard adolescents’ autonomous motivation during COVID-19 home-based learning

**DOI:** 10.1038/s41539-023-00153-2

**Published:** 2023-01-28

**Authors:** Ilona M. B. Benneker, Nikki C. Lee, Nienke van Atteveldt

**Affiliations:** 1 Section of Clinical Developmental Psychology & LEARN! Research Institute, Vrije Universiteit Amsterdam, Amsterdam, The Netherlands; 2 Mencia de Mendozalyceum, Breda, The Netherlands; 3 Department of Developmental Psychology, Utrecht University, Utrecht, The Netherlands

**Keywords:** Human behaviour, Education

## Abstract

During school closures throughout the COVID-19 pandemic, less support from peers and teachers may have required more autonomous motivation from adolescents. Little is known about factors that could shield against these negative effects. Driven by two influential motivational theories, we examined how mindset, feelings of school burnout and the three basic psychological needs of the self-determination theory, could predict changes in autonomous motivation when controlling for pre-pandemic levels of motivation. The results from a sample of Dutch adolescents (*M*_*age*_ = 14.63 years) and their parents (*M*_*age*_ = 48.65 years) showed that endorsing a growth mindset was positively associated with autonomous motivation during the school closures, while feelings of school burnout were negatively associated with autonomous motivation. Additionally, perceived parental autonomy support (i.e. a measure of the basic psychological need of autonomy) related to more autonomous motivation during home-based learning. Our findings highlight the personal and family factors that influence how adolescents respond to home-based learning and suggest ways to keep adolescents motivated and diminish possible negative consequences during future home-based learning situations.

## Introduction

The COVID-19 pandemic, that started in March 2020, required quick adaptations from Dutch adolescents when secondary schools closed and shifted to fully home-based learning for 11 weeks, before partially reopening. Consequently, teaching during these 11 weeks was fully online. This resulted in a substantial reduction in support from classmates and teachers, which may have increased the risk of adverse outcomes and required more autonomous motivation from the adolescent in order to continue to engage in their schoolwork. Moreover, the absence of school support amplified the influence of the home environment on adolescents’ motivation^1,2^ increasing the importance of parental support^3,4^. Recent studies investigating academic motivation during the COVID-19 pandemic have shown significant decreases in motivation^3,5–7^. Motivation is known to be an important determinant of academic success^8–10^ and a decrease in motivation could therefore have adverse consequences not just for the adolescents themselves, but also for society as a whole. As a consequence, it is important to understand both adolescents’ personal and family factors that could buffer against a potential loss of autonomous motivation during the challenging circumstances of the COVID-19 related school closures.

Adolescence, from the age of about ten to the early 20 s, may be a particularly vulnerable developmental period for the effects of school closures. It is a period characterized by significant cognitive, emotional and social changes^11–13^ that provide adolescents with particular advantages such as a greater flexibility in developing and adjusting autonomous (intrinsic) motivation^14^. During this period, the amount of time spent with parents decreases while the amount of time spent with peers increases^15^. However, due to school closures and the social restrictions of the COVID-19 pandemic, this normal transition of spending more time with peers and less with parents was curtailed. Much of the research conducted among adolescents during the first wave of the COVID-19 pandemic has focussed on the negative effects of this changed social situation on their mental health^16–18^. For example, a recent meta-analysis showed that adolescents were more likely to experience higher rates of depression and anxiety during the first period of home-based learning^19^. As school closures during the COVID-19 pandemic also drastically changed adolescents’ learning situation, it also increased the need for investigations on school-related factors, such as autonomous motivation. Autonomous motivation, which can be defined as engaging in behaviours with a full sense of volition and choice^20^, has previously been linked to academic achievement^8–10,21^ and is therefore important to investigate, particularly in early adolescents, as they still have a long period of schooling ahead. It is therefore very important to safeguard their motivation during secondary school.

A number of studies have focussed on motivation during the COVID-19 related school closures^6,22,23^ and a variety of predictors have been investigated. Some of these studies have focussed on negative predictors, such as anxiety and depression^3,24^, while others have focussed on predictors that positively relate to academic motivation and could therefore be protective factors, such as support from parents, peers and teachers^3,24–26^. In order to limit the possible long-term consequences of the pandemic’s impact, further research is needed on potential protective factors that could prevent the potential loss of autonomous motivation during the school closures. Here, we investigate potential personal and family protective factors based on two influential motivation theories; mindset theory^27^ and self-determination theory^28,29^.

According to Dweck’s mindset theory (1999), mindset can be defined as a disposition towards either the belief that intelligence and abilities are fixed, unchangeable traits or that they are more malleable and can be improved by hard work and practice. Adolescents with a more fixed mindset tend to avoid challenging problems and engage in maladaptive coping strategies^30^, while adolescents with a more growth mindset, think that success and failure are linked to effort and practice and not just to innate ability^31^. Adolescents with a more growth mindset display more adaptive strategies in the face of setbacks^32^, and may therefore show more resilient responses during the challenging circumstances of the school closures during the pandemic. Studies conducted before the school closures showed that adolescents who think that intelligence and skills are malleable, and are considered to have a growth mindset, tend to have higher autonomous (intrinsic) motivation^33–35^. A growth mindset might therefore shield against the possible risk of a decrease in autonomous motivation, since adolescents with a growth mindset are more able to handle the challenges of a period of home-based learning. Currently, a few studies have investigated the role of mindset^36,37^ and its relation to academic outcomes during the COVID-19 pandemic. These studies show that a growth mindset positively relates to learning engagement and may be a protective factor for feelings of academic stress. However, these studies investigated late adolescents. Our study focusses on early adolescents, as they are going through an important phase of social and cognitive changes that may impact their autonomous motivation^14,38^.

The potential protection of having a growth mindset might influence other factors, such as feelings of school burnout. School burnout is a continuous concept ranging from school related stress to a burnout and can be divided into three different dimensions, namely exhaustion at school, cynicism towards the meaning of schoolwork and a sense of academic inadequacy^39^. Previous investigations have shown that experiencing feelings of school burnout might have negative consequences on school related outcomes, such as lower academic achievement and a decline in motivation^21,40,41^. Furthermore, a recent investigation showed that adolescents with a growth mindset reported fewer feelings of school burnout^42^. More generally, having a growth mindset was associated with lower psychological distress and more effective coping with psychological distress^43^. It has therefore been suggested that adolescents with a growth mindset would show adaptive and resilient responses during school closures, which may have limited their experience of feelings of school burnout and consequently kept their autonomous motivation high. Therefore, we will consider whether feelings of school burnout could mediate the relationship between mindset and autonomous motivation during the period of home-based learning: a growth mindset may protect against motivation losses by limiting feelings of school burnout.

Another highly influential theory about predictors of autonomous motivation is self-determination theory. This theory has been widely utilized in understanding motivational outcomes in the educational context, and is therefore an important conceptual framework from which to study autonomous motivation in the drastically altered situation of home-based learning^28,29^. Self-determination theory proposes that humans have three fundamental needs that must be satisfied in order to achieve complete autonomous motivation: autonomy, relatedness and competence. The first two needs, autonomy and relatedness are affected by environmental inputs, such as autonomy support from parents^44–46^ and interaction with parents^47,48^ and became particularly relevant when the COVID-19 related school closures increased the importance of the home environment^1,3^. Adolescents need to feel that significant others, such as parents, support them and provide them with opportunities for choice and recognise them as an independent individual. In order to support the autonomy, parents should encourage their adolescents to act upon personally endorsed interests and at the same time take the adolescents’ perspective into account^29,49^. Previous pre-pandemic investigations have suggested that autonomy support from parents is positively related to adolescents’ autonomous motivation^50–55^. The second psychological need in self-determination theory is to feel relatedness to others. Adolescents should be accepted and feel connected to others^56^. During the home-based learning situation, interaction with parents could play an important role in satisfying adolescent’s need for relatedness. Lau & Leung (1992) for example showed in a prior investigation that a positive interaction between parents and children results in more attention to education and children who are more autonomously motivated. Other more recent investigations showed that positive parent-child interactions lead to more school engagement^48^. The focus on parent-child relationships is thus of great importance, since school closures might have drastically changed both the need for autonomy support from parents and the importance of a positive interaction between parents and adolescents.

The third basic need is task-related competence. Competence entails feeling effective and confident when pursuing an activity^56^ and is widely considered to be fundamental to motivation and achievement^57,58^. According to Bandura’s social cognitive theory, adolescents who feel competent are highly self-efficacious. This self-efficacy reflects how an individual views his or her abilities to perform a certain action, task or activity^59^. When schools closed, there was less direct support from teachers and classmates, and consequently, adolescents were required to complete their school work more independently. Within this context, individuals with high self-efficacy might have benefitted from their ability to work harder and persist longer when they encountered difficulties compared to those who doubted their abilities^13^. Pre-pandemic investigations indicated that adolescents with high self-efficacy showed high autonomous motivation^60–63^. Alivernini & Lucidi (2011) showed that self-efficacy positively impacts motivation over time. Therefore, this study investigated self-efficacy as the third basic psychological need hypothesized to predict autonomous motivation.

Here, we present data collected during the first and most intense part of the lockdown in the Netherlands (March–April 2020), a period when schools unexpectedly closed for the first time and education took place solely from home. We compare this to data collection in the month prior to this lockdown (i.e. pre-pandemic). The majority of studies into the impact of the pandemic on adolescents only use data collected during the pandemic, meaning they were unable to compare their data to a pre-pandemic measurement^3^. Other investigations use retrospective questions to mimic a pre-test measure^6^, meaning the data may have been impacted by incomplete recall of information or negatively biased answers. Therefore, an important contribution of our research is that is has true pre-pandemic data, collected one month before the school closures when there was no local threat yet. Uniquely, our sample consists of parent-adolescents dyads. This is of particular importance to for the first two basic psychological needs of the self-determination theory, as parents play an important role in supporting autonomy (perceived parental autonomy support) and relatedness (parent-adolescent interaction) during home-based learning. These parents and adolescents participated in our research both before the pandemic (February 2020) and again three weeks after the school closure.

In sum, we will first consider whether mindset protects adolescents from a possible potential decrease in autonomous motivation during home-based learning, when controlling for their pre-pandemic levels of autonomous motivation. Age and gender will be added as control variables, since younger adolescents showed a greater decrease in autonomous motivation than older adolescents during the pandemic^22^. We hypothesize that adolescents with a growth mindset will show higher levels of autonomous motivation during the home schooling period. We further predict that feelings of school burnout mediate the relationship between mindset and autonomous motivation, indicating that adolescents with a growth mindset will develop fewer symptoms of school burnout and might therefore be protected against a loss of autonomous motivation during the school closures when controlled for the pre-pandemic situation.

Next, we will investigate how the basic psychological needs, namely autonomy (measured as parental autonomy support) and relatedness (measured as positive parent-adolescent interaction), and competence (measured as self-efficacy) relate to the autonomous motivation of adolescents during the school closure, controlling for pre-pandemic levels of autonomous motivation. Gender of the parent and parental level of education will be added as control variables, the latter since parents with a higher level of education find themselves more capable of helping their children with their homework^1^. We hypothesize that high parental autonomy support, positive parent-child interaction and high self-efficacy will be positive predictors of autonomous motivation during home-based learning.

## Results

### Preliminary analysis of autonomous motivation

Table 1 presents the mean, standard deviations and correlations of the different measures taken before (February 2020) and during home-based learning (March-April 2020) from adolescents and their parents (*N* = 76 dyads).

**Table 1:** Mean, standard deviations and correlation of the different measurements parent-adolescent dyads (*N* = 76 parent-adolescent dyads).

	Mean	SD	1	2	3	4	5	6
1. Autonomous motivation (pre) (A)	2.80	0.62						
2. Autonomous motivation (post) (A)	2.68	0.62	0.59^a^					
3. Mindset (A)	4.62	0.73	0.40^a^	0.43^a^				
4. Feelings of school burnout (A)	3.17	1.10	−0.44^a^	-0.49^a^	-0.16^a^			
5. Perceived parental autonomy support (A)	3.21	0.43	0.31^a^	0.43^a^	0.16^a^	−0.54^a^		
6. Self-efficacy (A)	4.46	0.75	0.57^a^	0.40^a^	0.36^a^	−0.25^a^	0.24^a^	
7. Positivity of interaction (P)	4.32	0.69	0.11	0.09	0.15	0.02	0.42^a^	0.12

(A) measure reported by the adolescent, (P) measure reported by the parent.

^a^Correlation is significant at the 0.05 level.

We firstly examined how mindset and feelings of school burnout could predict changes in adolescents’ autonomous motivation during home-based learning when compared to before this period, using a stepwise linear regression model. As this analysis only required data reported by the adolescent, we used a larger sample (*N* = 97), which included the 76 adolescents who participated with their parents as well as an additional 21 adolescents whose parents did not complete (all) the questionnaires. Secondly, we examined how the three basic psychological needs, autonomy (measured as perceived parental autonomy support), relatedness (measured as positivity of parent-adolescent interaction) and competence (measured as self-efficacy) could explain autonomous motivation during the first school closures compared to the situation before. For this analysis, we used only the complete parent-adolescent dyads (*N* = 76 dyads).

A paired t-test showed that adolescents’ autonomous motivation significantly decreased during the lockdown (*M* = *2.68, SD* = *0.62*) compared to pre-measurement (*M* = *2.80, SD* = *0.45)*; (*t* (96) = 2.46, *p* = 0.017).

### Mindset and school burnout in relation to autonomous motivation

**Fig. 1:**
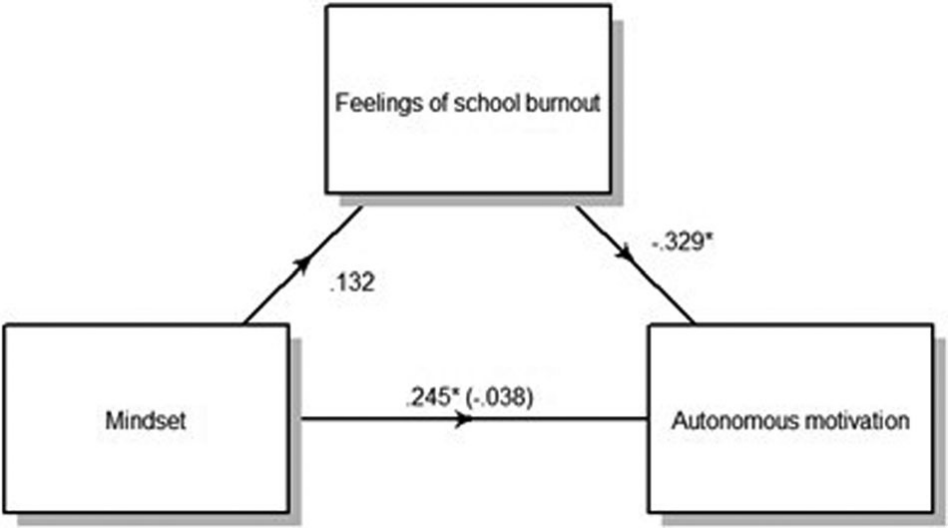
Standardized regression coefficients for the association between mindset and autonomous motivation as mediated by feelings of school burnout, controlling for gender, age and pre-measurement of autonomous motivation. *p < 0.025 (Bonferroni correction).

The first step of the first analysis based on the mindset theory (see Table 2) explained a significant 33% of the total variance on autonomous motivation during home-based learning. An effect of the pre-measurement of autonomous motivation (*β* = 0.59, *t*(93) = 5.84, *p* < 0.001) was shown, suggesting that adolescents with higher prior levels of autonomous motivation also had higher levels of autonomous motivation during the home-based learning period. In the second step, the predictor growth mindset explained a significant 8% of the additional variance of autonomous motivation during home-based learning when controlled for age, gender and pre-measurement of autonomous motivation. Specifically, a growth mindset contributed positively to autonomous motivation (*β* = 0.24, *t* (92) = 2.16, *p* = 0.020 (c’)). Within the mediation analysis, the regression of mindset on the mediator, feelings of school burnout, was not significant (*β* = 0.13, *t* (92) = 1.103, *p* = 0.275 (a)) (see Fig. 1). Feelings of school burnout, when controlling for gender, age, pre-measurement of autonomous motivation and mindset, did however significantly predict autonomous motivation (*β* = −0.17, *t* (91) = −2.798, *p* = 0.007 (b)). Unstandardized indirect effects were computed for each of 5000 bootstrapped samples. The indirect effect was −0.038 (c). We tested the significance of this indirect effect using bootstrapping procedures. The 95% confidence interval was computed by determining the indirect effects at the 2.5^th^ and 97.5^th^ percentiles and ranged from −0.12 to 0.03. The indirect effect was statistically non-significant (*p* = 0.251), showing that feelings of school burnout did not mediate the relation between mindset and autonomous motivation.

**Table 2:** Stepwise regression analysis of mindset and feelings of school burnout for autonomous motivation during home-based learning (*N* = 97 adolescents).

Variables	B	SE	β	t	p	R^2^	ΔR^2^
Step 1						0.333	0.364
Gender (adolescent)	−0.058	0.125	−0.047	−0.469	0.641		
Age (adolescent)	−0.020	0.030	−0.065	−0.642	0.523		
Autonomous motivation (pre)	0.844	0.145	0.589	5.838	<0.001^a^		
Step 2						0.408	0.370
Gender (adolescent)	−0.045	0.121	−0.037	−0.378	0.668		
Age (adolescent)	0.005	0.032	0.017	0.157	0.706		
Autonomous motivation (pre)	0.723	0.151	0.504	4.776	<0.001^a^		
Mindset	0.212	0.098	0.245	2.158	0.020^a^		
Step 3						0.472	0.427
Gender (adolescent)	0.067	0.126	0.053	0.524	0.602		
Age (adolescent)	0.033	0.031	0.113	1.060	0.293		
Autonomous motivation (pre)	0.503	0.169	0.344	2.971	0.004^a^		
Mindset	0.254	0.095	0.294	2.662	0.001^a^		
Feelings of school burnout	−0.174	0.062	−0.329	−2.798	0.007^a^		

Gender is coded as 1 = male and 2 = female. *F*-statistics: Step 1: *F* (3, 93) = 12.04, *p* < 0.001; Step 2: *F* (4, 92) = 10.70, *p* < 0.001; Step 3: *F* (5, 91) = 10.55, *p* < 0.001.

^a^Correlation is significant at the 0.025 level (Bonferroni correction).

### The self-determination theory in relation to autonomous motivation

The first step of the second analysis on the three basic psychological needs (see Table 3) explained a significant 41% of the total variance in adolescents’ autonomous motivation during home-based learning. Equally to the previous stepwise regression analysis of adolescents’ autonomous motivation, a positive effect of the pre-measurement of autonomous motivation (*β* = 0.63, *t*(72) = 6.27, *p* < 0.001) was shown. In the second step, we added perceived parental autonomy support, self-efficacy and positivity of parent-adolescent interaction, which together explained a significant 8% of the additional variance after controlling for gender and level of education of the parent and pre-measurement of adolescents’ autonomous motivation. There was a significant positive effect of gender (β = 0.24, *t* (69) = 2.40, *p* = 0.020) and perceived parental autonomy support (*β* = 0.28, *t* (69) = 2.41, *p* = 0.019). This suggests that the involvement of mothers tended to relate to higher levels of autonomous motivation among adolescents during the home-based learning period than the involvement of fathers. Furthermore, adolescents who perceived their parents as supporting their autonomy had higher levels of autonomous motivation compared to those who perceived their parents as less supportive of their autonomy.

**Table 3:** Stepwise regression analysis of three basic psychological needs of the self-determination theory on autonomous motivation during home-based learning (*N* = 76 parent-adolescent dyads).

Variables	B	SE	β	t	p	R^2^	DR^2^
Step 1						0.412	0.384
Gender (parent)	0.311	0.140	0.218	2.223	0.030		
Level of education (parent)	0.043	0.090	0. 047	0.471	0.640		
Autonomous motivation (pre) (A)	0.879	0.140	0.879	0.6274	<0.001^a^		
Step 2						0.493	0.433
Gender (parent)	0.368	0.154	0.244	2.395	0.020^a^		
Level of education (parent)	-0.046	0.102	−0.047	−0.454	0.652		
Autonomous motivation (pre) (A)	0.679	0.172	0.491	3.953	<0.001^a^		
Perceived parental autonomy support (A)	0.386	0.161	0.275	2.405	0.019^a^		
Positivity of interaction (P)	−0.09	0.103	−0.099	−0.899	0.372		
Self-efficacy (A)	0.105	0.098	0.133	1.068	0.290		

(A) is measurement taken from the adolescent, (P) is measurement taken from the parent; Gender is coded as 1 = male and 2 = female; F-statistics; Step 1 F (3, 93) = 14.69, *p* < 0.001; Step 2 F (6, 90) = 8.256, *p* < 0.001.

^a^Regression is significant at the 0.025 level (Bonferroni correction).

## Discussion

Guided by two influential motivational theories, our research aimed to investigate how mindset and school burnout and the three basic psychological needs defined by the self-determination theory, could predict changes in autonomous motivation during home-based learning when controlled for levels of autonomous motivation before the pandemic.

We found that mindset was positively related to autonomous motivation during home-based learning, while controlling for the pre-pandemic situation. We found no mediation effect for feelings of school burnout on this relationship, but we did find that feelings of school burnout were negatively related to autonomous motivation during the school closures. Regarding the three basic psychological needs described by the self-determination theory, we found that perceived parental autonomy support was positively related to autonomous motivation during home-based learning. We did not find an effect of positivity of interaction or competence. We discuss each of these findings and their implications in more detail below.

With respect to the mindset theory^27^, our results showed that having a growth mindset was positively related to autonomous motivation during the period of home-based learning. As we controlled levels of pre-pandemic motivation, this indicates that adolescents with a growth mindset were better at maintaining their autonomous motivation during the sudden switch from school to home-based learning. This is in line with what we predicted based on studies carried out before the pandemic, which showed that adolescents with a growth mindset had higher levels of motivation^33–35^. This may mean that, even in adverse circumstances, those with a growth mindset show consistent interest and resilience with regards to their learning tasks, potentially by exerting more mastery-oriented strategies^32^. They may therefore be more able to cope with their new learning context, in which they are more responsible for their school work and dealing with any setbacks they encounter. A growth mindset may therefore serve as an important protective factor for preventing a decline in adolescents’ autonomous motivation during home-based learning. This underlines the importance of the development and implementation of interventions that could foster a growth mindset. Although the effectiveness of growth mindset interventions has been challenged^64^, there has also been much empirical research in support of using these interventions to improve academic achievement, as well as motivation^65,66^. Yeager et al. (2019) developed an online growth mindset intervention that teaches adolescents that their intellectual abilities can be improved. Their results showed that grades increased in particular among lower-achieving students. An important aspect of this intervention is that it took place online, which makes it in particularly useful for implementation during possible future school closures. Whereas Yeager et al. (2019) focussed more on academic achievement, Sarrasin et al. (2018) also looked at motivational outcomes in their meta-analysis. In line with the results on academic achievement, Sarrasin et al. (2018) also found a positive effect of growth mindset interventions on motivation, benefitting at-risk students more than not-at-risk students. Lastly, a very recent study shows that short online mindset interventions can be beneficial for COVID-19 related well-being^67^.

Our results did not show a mediation effect of feelings of school burnout on the relationship between mindset and autonomous motivation during home-based learning. Feelings of school burnout however did predict autonomous motivation. Adolescents’ feelings of school burnout, such as being exhausted by the school work or feeling inadequate about the school work, could possibly make adolescents feel insecure about their abilities^41^ and this could in turn decrease their autonomous motivation^40^. This highlights the importance of carefully monitoring adolescents’ school burnout symptoms during home-based learning as it negatively influences autonomous motivation. This monitoring could be accompanied by preventative measures. For example, previous research shows that focussing on self-regulation skills, such as grit and academic buoyancy, as well as on social skills, such as negotiation and resolving conflicts, can help adolescents to handle academic stress and prevent feelings of school burnout^68,69^.

From the perspective of the basic psychological needs described by the self-determination theory, we found that autonomy (measured as perceived parental autonomy support) was positively related to autonomous motivation during the school closures. Adolescents autonomous motivation appear to benefit from parental support of their autonomy. Parent can do this by taking the perspective of the adolescent into account and encouraging them to act upon their own values and interests, while also providing structure and boundaries^29,49^. During periods of home-based learning there will be increased opportunities for parents to support their adolescents’ independence and show them that they accept and trust their choices, which in turn influence theirs adolescents’ autonomous motivation. An important first step during a period of home-based learning will therefore be to make parents aware of the important role they have in supporting autonomy in their adolescents.

We did not find an effect of relatedness (measured as positivity of parent-adolescent interaction) nor did we find an effect of competence (measured as self-efficacy) on autonomous motivation during the first home-based learning period compared to pre-pandemic levels. It is possible however, since the parent-adolescent interaction was reported only by the parent, that there is a discrepancy between the perception of the adolescent and the parent, resulting in a non-significant relationship. It is possible that adolescents’ perceived positivity of the parent-adolescent interaction would have provided us with different results. Additionally the results on self-efficacy were unexpected. Although self-efficacy was positively related to autonomous motivation pre-pandemic as well as during home-based learning (see Table 1), it did not seem to act as a protective factor against a loss in motivation.

A number of limitations should be noted with regards to the current study. First, the adolescents and parents who participated in this study were mostly highly educated and came from an affluent area in the southern part of the Netherlands. It may therefore be difficult to generalize our findings to other (sub)groups. An initial study in the Netherlands showed that children from advantaged backgrounds generally receive much more parental support than children from more disadvantaged backgrounds^1^, suggesting that there may be differences between social groups. It is therefore important, during future periods of home-based learning, to also focus on other, less affluent parts of the Netherlands and other countries.

Second, an advantage of our dyadic data (data from both parents and their adolescents) is that it was possible to measure (self-reported) interaction between parents and their adolescents and this gives us more insight into this interaction compared to using only measurements from either the adolescent or parent. However, a disadvantage of these dyadic-data is that the recruitment of both parents and their adolescents presents additional practical hurdles relative to studying individuals (for example both the parents and their adolescent have to respond within a certain time period). This contributed to the smaller sample size for our second analysis.

Third, in our investigation we are unable to provide causal inferences from the data due to their observational nature. While the specific circumstances of the COVID-19 pandemic will hopefully not reoccur, it is likely that there will be other challenging educational situations in the future. Therefore, it would be useful for future studies to use experimental research designs to make informative inferences about the causal mechanisms behind autonomous motivation during home-based learning.

Fourth, to get a more in-depth view on adolescent resilience factors and the psychological needs of adolescents, future studies should go beyond self-report and combine questionnaires with observations or interviews with parents and their adolescent(s) in order to understand how parental interaction might influence academic outcomes in adolescents.

In sum, this study examined predictors of autonomous motivation during home-based learning informed by two important motivational theories: mindset^27^ and the three basic psychological needs described by self-determination theory^29^. We found several protective factors that may have important implications for current or future instances of home-based learning. Having a growth mindset seems to be a protective factor for a decrease in autonomous motivation. Our results also show that adolescents with high levels of school burnout show lower levels of autonomous motivation in adverse circumstances, such as the unexpected change from school-based learning to home-based learning. Additionally, our results provided evidence for the positive role of perceived parental autonomy support in autonomous motivation in adolescents. These findings have useful implications, such as highlighting the importance of deploying (online) growth mindset interventions for adolescents, as well as providing insights into the importance of parents supporting autonomy in adolescents during home-based learning. These may help to keep adolescents motivated and to diminish possible negative consequences during future school closures or other instances in which home schooling takes place.

## Methods

### Participants

Participants were Dutch adolescents and one of their parents from two different secondary schools in the region of Breda in the Netherlands. The researchers contacted schools interested in participating in scientific research to ask for permission to send recruitment information to adolescents and their parents. Schools that agreed to participate were asked to forward an email with information about the research project to all parents. An initial 130 adolescents participated with one of their parents. Of these adolescents eight (6%) missed the pre-measurement, while 25 (19%) missed the measurement during the school closure. These 33 adolescents were therefore excluded from further analysis, resulting in sample of 97 adolescents. Of the 130 parents, five (4%) of the parents missed the pre-measurement, while 21 parents (16%) missed the measurement during the school closure. When combining the adolescent and parent data there were a total of 49 parent-adolescent dyads with one missing measurement and five parent-adolescent dyads with multiple missing measurements. Dyads with missing data were excluded from further analysis, resulting in a total of 76 complete parent-adolescent dyads.

For the first analysis we used only data from the adolescents and we could hence include a larger sample in this analysis, since these measurements were independent from the measurements of the parents. A total of 97 adolescents were included in the first analysis. For the second analysis we combined data from adolescents and their parents, and therefore used the 76 parent-adolescent dyads.

The mean age of the adolescents (58% female) was 14.45 years (SD = 2.01) (see Table 4 for descriptives), the mean age of the parents (76% female) was 48.67 years (SD = 4.60).

**Table 4:** Descriptive statistics of the adolescents and parents: age, gender and level of education.

	Adolescents (*n* = 97)	Parents (*n* = 76)
Gender (f: m)	56: 41	58: 18
Age		
Mean (SD)	14.45 years (2.01)	48.67 years (4.60)
Range	11.50–19.83 years	34.75–62.67 years
Level of education		Highest level of education (%)
Primary school		0.8
Secondary school		4.0
MBO		5.6
HBO		48.4
WO		39.5
Other		1.6^a^

The Dutch schooling system after secondary school is divided into MBO (middelbaar beroepsonderwijs), which is focussed on vocational training, HBO which focussed on general higher education and WO (wetenschappelijk onderwijs, i.e., university). HBO education focuses on vocational training in subjects such as nursing and teaching, whereas WO education offers higher level programs at research universities, such as medicine and law.

^a^This level of education was excluded from the analysis.

### Procedure

Prior to the study, adolescents and their parents gave written informed consent and all procedures were approved by the Vrije Universiteit Amsterdam, Faculty of Behavioural and Human Movement sciences ethics committee. This investigation is part of a larger study on fluctuations in ability beliefs and motivation over time in Dutch adolescents. In February 2020, adolescents completed a questionnaire at home (via their mobile device) on autonomous motivation, mindset and self-efficacy. Demographic measures, including age and gender, were also included in the questionnaire. After the first three weeks of the school closure (March–April 2020), adolescents received an additional questionnaire which consisted of questions on autonomous motivation, feelings of school burnout and perceived parental autonomy support. Parents answered questions (via their mobile device) on gender, age and level of education (in February 2020) and they received an additional questionnaire after the first three weeks of school closure (March–April 2020) asking them about the positivity of interaction with their adolescent during the past three weeks. Adolescents and their parents had three days to complete the additional questionnaire.

### Measures

#### Autonomous motivation (reported by adolescents)

Two subscales, intrinsic and identified regulation, of the short version of the Academic Self-Regulation Questionnaire (SRQ-A) were used^70–72^. Both subscales consist of three items. An example of an item from the intrinsic regulation subscale is ‘I do my classwork because it is fun’ and an example from the identified regulation subscale is ‘I do my classwork because I want to learn new things’. A translated Dutch version of the SRQ-A was used, which was back-translated by a native English speaker. Answers were scored on a Likert-scale from 1 to 4 where 1 meant ‘not at all true’ and 4 ‘very true’. The two separate subscales (intrinsic and identified regulation) are considered the two most autonomous forms of motivation and highly correlate. We therefore used them together to create a combined autonomous motivation subscale^73^, as has been done in previous investigations^74,75^. These prior investigations with adolescents have verified the validity of this autonomous motivation measure based on these two subscales in different adapted versions of the Self-Regulation Questionnaires of Ryan & Connell (1989). Results showed adequate to excellent fit^74,75^. The average score on the items on the two separate subscales was calculated and these were averaged into a single score for autonomous motivation, with higher scores indicating higher levels of autonomous motivation. Internal consistency was measured using Cronbach’s alpha (a) for the pre-measurement (*a* = 0.69) and the post measurement (*a* = 0.83) showing acceptable to good reliability.

### Mindset (reported by adolescents)

Mindset, also referred to as (general) ability beliefs, was measured by using the Implicit theories of Intelligence Scale of Dweck (1999)^27^ adapted by de Castella & Byrne (2015)^76^ and adapted to Dutch by van Aalderen–Smeets et al. (2019)^77^. The scale consists of 4 entity theory items and 4 incremental theory items. Answers were scored on a Likert-scale from 1 to 6 in which 1 meant ‘strongly disagree’ and 6 ‘strongly agree’. The four entity theory items were reverse scored and the mean of the eight items was calculated. A higher score meant having a stronger growth mindset, while a lower score meant having a stronger fixed mindset. An example of a growth mindset statement is ‘Regardless of my current intelligence level, I think I have the capacity to change it quite a bit’ and an example of a fixed mindset statement is ‘To be honest, I don’t think I can really change how intelligent I am’. Validity of the Dutch version of the (general) ability beliefs of intelligence scale (mindset) has been checked in previous work and the (combined entity (i.e. fixed) and incremental (i.e. growth)) scale was found to be of good validity^77^. Cronbach’s alpha for internal consistency showed good reliability (*a* = 0.89).

### Feelings of school burnout (reported by adolescents)

The school burnout inventory (SBI) measures symptoms of burnout of adolescents in a school related context^39^. It consists of three subscales; exhaustion (four items), cynicism (three items) and feelings of inadequacy (two items). Three example items from the subscales exhaustion, cynicism and feelings of inadequacy are respectively ‘I feel overwhelmed by my schoolwork’, I feel a lack of motivation in my schoolwork and often think about giving up, ‘I used to have higher expectations of my schoolwork than I do now’. A translated Dutch version of the SBI was used, which was back-translated by a native English speaker. Answers were scored on a Likert-scale from 1 to 6 in which 1 meant ‘strongly disagree’ and 6 ‘strongly agree’. In line with the third model of Salmela-Aro (2009), we measured overall school burnout score as an average score of the three different first-order factors: exhaustion, cynicism and feelings of inadequacy. Then the average of these three factors was calculated, which reflected a second-order overall school burnout score with higher scores indicating higher feelings of school burnout^39^. The validity of this scale was checked in a prior investigation and the scale was found to be adequate^39^. For internal consistency, we measured Cronbach’s alpha which showed good reliability (*a* = 0.88).

### Self-efficacy (reported by adolescents)

Self-efficacy was assessed by using a 5-item scale from the Patterns of adaptive learning scales^78^ and adapted to Dutch by van Aalderen-Smeets et al. (2019)^77^. Responses were made on a 6-point Likert scale ranging from 1 (strongly disagree) to 6 (strongly agree). An example of an item is ‘I can do almost all the work in class if I don’t give up’. The average of the five items was calculated, with higher scores indicating higher self-efficacy. Validity of the Dutch version of this scale as checked in previous studies and the self-efficacy scale was found to be of good validity^77^. Cronbach’s alpha for internal consistency showed good reliability (*a* = 0.76).

### Perceived parental autonomy support (reported by adolescents)

The child version of the Parent Child Interaction – Revised scale was used. This is a Dutch questionnaire that consists of 30 items and refers to both interpersonal behaviour and interpersonal feelings^79^. The questionnaire consists of two subscales. The authority subscale contains nine items and refers to parental skills that establish a certain hierarchical relationship between parents and their children, which means that adolescents should feel that their parents are respectful, within certain boundaries, towards the choices their adolescents make. An example of an item of this subscale is ‘When I have a problem I ask my father / mother for advice’. Three items of this subscale were reverse scored. The other subscale, acceptance, contains 21 items and refer to parents’ unconditional acceptance and involvement with their child and is characterized by encouragement of positive behaviour and willingness to share emotions with the child. An example of an item of this subscale is ‘My father / mother listens to me when I want to talk to him/her’. Ten items of this subscale were reverse scored. The average of the scores from the items of the two different subscales was calculated. According to previous investigations, these two subscales combined can be used to measure to what extent adolescents perceive their parents to be supportive of their autonomy^29,80,81^. Higher scores indicate perceiving more parental autonomy support. The validity of the use of these test scores for this scale has been checked in a previous investigation^79^. In this previous study, this measure was shown to have satisfactory validity for measuring parent-child interaction in typically developing adolescents such as those investigated in our sample. Cronbach’s alpha for internal consistency showed excellent reliability (*a* = 0.94).

### Positivity of interaction (reported by parents)

Parents were asked with two separate items to what extent the interaction with their adolescent in the first three weeks of home-based learning was positive or negative. Responses were made on a 5-point Likert scale ranging from 1 (not at all) to 5 (very much) for both positive and negative interaction. The scale with negative interaction was reverse scored. The two scales were combined and the average was calculated, with higher scores indicating that the parent-adolescent interaction was indicated by the parents as more positive.

### Analysis

To test whether autonomous motivation during home-based learning is predicted by the factors hypothesized based on (1) mindset theory and (2) self-determination theory, when controlled for the within-subject pre-pandemic levels of autonomous motivation, we conducted two stepwise multiple regression analysis. All assumptions were checked and found to be satisfactory.

Step 1 of the first model, with autonomous motivation (see Table 2) as outcome variable consisted of age, gender and the pre-pandemic measurements of autonomous motivation as control variables. In step 2 mindset was added as a predictor and in step 3 feelings of school burnout were analysed as a mediator between mindset and autonomous motivation (see Fig. 1 and step 3 of Table 2).

Step 1 of the second model, with autonomous motivation (see Table 3) as outcome variable consisted of gender and level of education of the parent and the pre-pandemic measurements of autonomous motivation as control variables. Next, step 2 introduced autonomy (measured as perceived parental autonomy support), relatedness (measured as positive parent-adolescent interaction) and competence (measured as self-efficacy) as predictors. All analysis were performed using R version 4.0.3^82^.

The two separate analyses have the same outcome variable (autonomous motivation during home-based learning). This increases the chance of committing type I errors. In order to reduce this chance, we adopted the Bonferroni correction for the two regression analysis, i.e. alpha = 0.05/2 = 0.025. A power analysis for the first regression analysis was conducted using G*Power^83^ for sample size estimation, based on *N* = 97. The effect size in this study was 0.2, considered to be medium using Cohen’s (1988) criteria. With a significance of α = 0.025 and power = 0.8, the minimum sample size needed for this effect size is *N* = 70 for the regression analysis relating mindset and feelings of school burnout to autonomous motivation controlling for age, gender and pre-measurement of autonomous motivation. With our sample of *N* = 97, we have enough power to detect at least medium sized effects. Power analysis for the second regression analysis with effect size 0.2 for a sample size estimation based on *N* = 76, *α* = 0.025 and power = 0.8, demonstrated that a minimum sample size of *N* = 74 is needed, so with our sample size of N = 76 we can at least detect medium sized effects.

### Reporting summary

Further information on research design is available in the Nature Research Reporting Summary linked to this article.

## Supplementary information

Reporting Summary

## Data Availability

Data may be accessed for research purposes, upon request and in line with current privacy regulations.
