# Supplementary material for: Mindset and perceived parental support of autonomy safeguard adolescents’ autonomous motivation during COVID-19 home-based learning
Source: NPJ Sci Learn. 2023 Jan 28;8:4. doi: 10.1038/s41539-023-00153-2 (PMC9883818; doi:10.1038/s41539-023-00153-2)
Supplement: Supplementary file 1 — Reporting Summary [file 41539_2023_153_MOESM1_ESM.pdf]

nature portfolio

Corresponding author(s): Ilona Benneker

Last updated by author(s): Dec 30, 2022

Reporting Summary

Nature Portfolio wishes to improve the reproducibility of the work that we publish. This form provides structure for consistency and transparency in reporting. For further information on Nature Portfolio policies, see our Editorial Policies and the Editorial Policy Checklist.

Statistics

For all statistical analyses, confirm that the following items are present in the figure legend, table legend, main text, or Methods section.

n/a	Confirmed	
☐	☒	The exact sample size (*n*) for each experimental group/condition, given as a discrete number and unit of measurement
☐	☒	A statement on whether measurements were taken from distinct samples or whether the same sample was measured repeatedly
☐	☒	The statistical test(s) used AND whether they are one- or two-sided<br>*Only common tests should be described solely by name; describe more complex techniques in the Methods section.*
☐	☒	A description of all covariates tested
☐	☒	A description of any assumptions or corrections, such as tests of normality and adjustment for multiple comparisons
☐	☒	A full description of the statistical parameters including central tendency (e.g. means) or other basic estimates (e.g. regression coefficient) AND variation (e.g. standard deviation) or associated estimates of uncertainty (e.g. confidence intervals)
☐	☒	For null hypothesis testing, the test statistic (e.g. F, t, r) with confidence intervals, effect sizes, degrees of freedom and P value noted<br>*Give P values as exact values whenever suitable.*
☒	☐	For Bayesian analysis, information on the choice of priors and Markov chain Monte Carlo settings
☒	☐	For hierarchical and complex designs, identification of the appropriate level for tests and full reporting of outcomes
☐	☒	Estimates of effect sizes (e.g. Cohen's *d*, Pearson's *r*), indicating how they were calculated

Our web collection on statistics for biologists contains articles on many of the points above.

Software and code

Policy information about availability of computer code

Data collection	The output for this paper was generated using Qualtrics software, Version XM of Qualtrics. Copyright © 2020 Qualtrics. Qualtrics and all other Qualtrics product or service names are registered trademarks or trademarks of Qualtrics, Provo, UT, USA. https://www.qualtrics.com
Data analysis	All analysis were performed using R version 4.0.3. R Core Team (2020). R: A language and environment for statistical computing. R Foundation for Statistical Computing, Vienna, Austria. URL https://www.R-project.org/

For manuscripts utilizing custom algorithms or software that are central to the research but not yet described in published literature, software must be made available to editors and reviewers. We strongly encourage code deposition in a community repository (e.g. GitHub). See the Nature Portfolio guidelines for submitting code & software for further information.

Data

Policy information about availability of data

All manuscripts must include a data availability statement. This statement should provide the following information, where applicable:

- Accession codes, unique identifiers, or web links for publicly available datasets
- A description of any restrictions on data availability
- For clinical datasets or third party data, please ensure that the statement adheres to our policy

Data may be accessed for research purposes, upon request and in line with current privacy regulations.

Human research participants

Policy information about studies involving human research participants and Sex and Gender in Research.

Reporting on sex and gender	Both adolescents and parent self-reported their gender. Participants could choose to report their gender as male or female, or could choose not to answer the question. Adolescents' gender was used as a control variable in our first analysis with only adolescents as participants. In the second analysis we used parents' gender as a control variable. In the informed consent, we stated that 'researcher will preserve confidentiality by anonymizing responses before publication in e.g. a scientific journal'. A total of 97 adolescents were included in the analyses: 56 female and 41 male. A total of 76 parents were included in the analyses: 58 female and 18 male.
Population characteristics	Participants were Dutch adolescents from two different secondary schools in the region of Breda in the Netherlands. The average age of the adolescents was 14.63 years; the average age of the parents was 48.6 years.
Recruitment	The researchers contacted schools interested in participating in scientific research to ask for permission to send recruitment information to adolescents and their parents. Schools that agreed to participate were asked to forward an email with information about the research project to all parents. The adolescents and parents who participated in this study were mostly highly educated and came from an affluent area in the southern part of the Netherlands. It may therefore be difficult to generalize the findings to other (sub)groups.
Ethics oversight	Vrije Universiteit Amsterdam, Faculty of Behavioural and Human Movement sciences ethics committee

Note that full information on the approval of the study protocol must also be provided in the manuscript.

Field-specific reporting

Please select the one below that is the best fit for your research. If you are not sure, read the appropriate sections before making your selection.

☐ Life sciences ☒ Behavioural & social sciences ☐ Ecological, evolutionary & environmental sciences

For a reference copy of the document with all sections, see nature.com/documents/nr-reporting-summary-flat.pdf

Behavioural & social sciences study design

All studies must disclose on these points even when the disclosure is negative.

Study description	This investigation is part of a larger descriptive study on fluctuations in ability beliefs and motivation over time in Dutch adolescents. All data collected was quantitative. In February 2020, adolescents completed a questionnaire at home (via their mobile device) on autonomous motivation, mindset and self-efficacy. Demographic measures, including age and gender, were also included in the questionnaire. After the first three weeks of the school closure (March-April 2020), adolescents received an additional questionnaire which consisted of questions on autonomous motivation, feelings of school burnout and perceived parental autonomy support. Parents answered questions (via their mobile device) on gender, age and level of education (in February 2020) and they received an additional questionnaire after the first three weeks of school closure (March-April 2020) asking them about the positivity of interaction with their adolescent during the past three weeks. Adolescents and their parents had three days to complete the additional questionnaire.
Research sample	Participants were Dutch adolescents and one of their parents from two different secondary schools in the region of Breda in the Netherlands. The average age of the adolescents was 14.63 years (56 female / 41 male). The average age of the parents was 48.6 years (58 female / 18 male). This investigation focusses on early adolescents, since they still have a long period of schooling ahead and it is therefore important to safeguard their motivation during secondary school. The adolescents and parents who participated in this study were mostly highly educated and came from an affluent area in the southern part of the Netherlands. It may therefore be difficult to generalize the findings to other (sub)groups.
Sampling strategy	The researchers contacted schools interested in participating in scientific research to ask for permission to send recruitment information to adolescents and their parents. Schools that agreed to participate were asked to forward an email with information about the research project to all parents. Adolescents and one of their parents who choose to participate followed a link to give informed consent and to choose to participate in the study. There were no exclusion criteria.<br><br>The current investigation is part of a larger longitudinal study with a strong emphasis on motivation and was designed before the pandemic. The first wave of data collection was completed before the pandemic started (Feb-March 2020). The rise of COVID-19 pandemic meant that the second wave was conducted during the first period of school closures and other COVID-related restrictions. This meant that the second part of the study was designed post-hoc and resulted from an extremely unexpected situation. As a result the design was constrained by the measures that were selected pre-pandemic. Power analysis, using G*Power 3, with an effect size of .2 (medium), alpha = .025 and power = .8 for the first research question with 5 predictor variables, the minimum sample size is N = 70. So, with our sample of N = 97 adolescents, we have enough power to detect at least medium sized effects. For the second analysis, power analysis with an effect size of .2, alpha = .025 and power = .8 for the second research question with 6 predictor variables, the minimum sample size is N = 74. This also shows we also had sufficient power to detect at least medium sized effects

	with our 76 parent-adolescent dyads.
Data collection	In February 2020, adolescents completed a questionnaire at home (via their mobile device) on autonomous motivation, mindset and self-efficacy. Demographic measures, including age and gender, were also included in the questionnaire. After the first three weeks of the school closure (March-April 2020), adolescents received an additional questionnaire which consisted of questions on autonomous motivation, feelings of school burnout and perceived parental autonomy support. Parents answered questions (via their mobile device) on gender, age and level of education (in February 2020) and they received an additional questionnaire after the first three weeks of school closure (March-April 2020) asking them about the positivity of interaction with their adolescent during the past three weeks. Adolescents and their parents had three days to complete the additional questionnaire.
Timing	Feb 2020 (pre-measurement) and March-April 2020 (covid-measurement)
Data exclusions	An initial 130 adolescents participated with one of their parents. Of these adolescents eight (6%) missed the pre-measurement, while 25 (19%) missed the measurement during the school closure. These 33 adolescents were therefore excluded from further analysis, resulting in sample of 97 adolescents. Of the 130 parents, five (4%) of the parents missed the pre-measurement, while 21 parents (16%) missed the measurement during the school closure. When combining the adolescent and parent data there were a total of 49 parent-adolescent dyads with one missing measurement and five parent-adolescent dyads with multiple missing measurements. Dyads with missing data were excluded from further analysis, resulting in a total of 76 complete parent-adolescent dyads.
Non-participation	No participants dropped out.
Randomization	Participants were not allocated into random groups.

Reporting for specific materials, systems and methods

We require information from authors about some types of materials, experimental systems and methods used in many studies. Here, indicate whether each material, system or method listed is relevant to your study. If you are not sure if a list item applies to your research, read the appropriate section before selecting a response.

Materials & experimental systems

n/a	Involved in the study	
☒	☐	Antibodies
☒	☐	Eukaryotic cell lines
☒	☐	Palaeontology and archaeology
☒	☐	Animals and other organisms
☒	☐	Clinical data
☒	☐	Dual use research of concern

Methods

n/a	Involved in the study	
☒	☐	ChIP-seq
☒	☐	Flow cytometry
☒	☐	MRI-based neuroimaging
